# Detection of Mixed Infection from Bacterial Whole Genome Sequence Data Allows Assessment of Its Role in *Clostridium difficile* Transmission

**DOI:** 10.1371/journal.pcbi.1003059

**Published:** 2013-05-02

**Authors:** David W. Eyre, Madeleine L. Cule, David Griffiths, Derrick W. Crook, Tim E. A. Peto, A. Sarah Walker, Daniel J. Wilson

**Affiliations:** 1 Nuffield Department of Clinical Medicine, University of Oxford, John Radcliffe Hospital, Oxford, United Kingdom; 2 NIHR Oxford Biomedical Research Centre, John Radcliffe Hospital, Oxford, United Kingdom; 3 Department of Statistics, University of Oxford, Oxford, United Kingdom; 4 Medical Research Council, Clinical Trials Unit, London, United Kingdom; 5 Wellcome Trust Centre for Human Genetics, Oxford, United Kingdom; Imperial College London, United Kingdom

## Abstract

Bacterial whole genome sequencing offers the prospect of rapid and high precision investigation of infectious disease outbreaks. Close genetic relationships between microorganisms isolated from different infected cases suggest transmission is a strong possibility, whereas transmission between cases with genetically distinct bacterial isolates can be excluded. However, undetected mixed infections—infection with ≥2 unrelated strains of the same species where only one is sequenced—potentially impairs exclusion of transmission with certainty, and may therefore limit the utility of this technique. We investigated the problem by developing a computationally efficient method for detecting mixed infection without the need for resource-intensive independent sequencing of multiple bacterial colonies. Given the relatively low density of single nucleotide polymorphisms within bacterial sequence data, direct reconstruction of mixed infection haplotypes from current short-read sequence data is not consistently possible. We therefore use a two-step maximum likelihood-based approach, assuming each sample contains up to two infecting strains. We jointly estimate the proportion of the infection arising from the dominant and minor strains, and the sequence divergence between these strains. In cases where mixed infection is confirmed, the dominant and minor haplotypes are then matched to a database of previously sequenced local isolates. We demonstrate the performance of our algorithm with *in silico* and *in vitro* mixed infection experiments, and apply it to transmission of an important healthcare-associated pathogen, *Clostridium difficile*. Using hospital ward movement data in a previously described stochastic transmission model, 15 pairs of cases enriched for likely transmission events associated with mixed infection were selected. Our method identified four previously undetected mixed infections, and a previously undetected transmission event, but no direct transmission between the pairs of cases under investigation. These results demonstrate that mixed infections can be detected without additional sequencing effort, and this will be important in assessing the extent of cryptic transmission in our hospitals.

## Author Summary

Traditionally, outbreaks of infectious diseases are investigated by considering contact between cases and their exposure to possible sources of infection. This can be enhanced by using the genetic fingerprint of bacteria to rule out transmission between cases infected with unrelated strains. However, in some cases patients are infected with more than one strain of the same species of bacteria. This is known as mixed infection. Using current methods usually only one strain of bacteria is analysed, so transmission might be ruled out wrongly if there is a mixed infection. We developed a method that exploits new high-resolution genetic fingerprinting in bacteria to detect patients that are infected with multiple strains of the same bacterial species. We investigated the important healthcare-associated infection *Clostridium difficile*, revealing previously undetected mixed infections, and identifying a previously undetected transmission event. By interrogating a database of bacterial strains, our method deduced the mixed strain types, which we showed were not compatible with direct transmission among the patients under investigation. Our method can improve the sensitivity of outbreak investigation across different types of bacteria, which will ultimately help to reduce transmission in hospitals and the community.

## Introduction

Whole genome sequencing (WGS) offers the prospect of high precision investigation of infectious disease outbreaks [1], [2]. Close genetic relationships between organisms isolated from different infected cases suggest transmission is a strong possibility, whereas transmission between cases with genetically distinct isolates can be excluded. WGS has been successfully applied to several high profile national outbreaks, in particular the *Escherichia coli* outbreak in Germany [3]–[5], and cholera outbreak in Haiti [6]. The advent of rapid benchtop sequencing technology allows WGS to be applied in clinically relevant timescales to local outbreaks, for example those caused by the important healthcare-associated pathogens *Clostridium difficile* and MRSA [7], [8]. The increased resolution offered by WGS allows isolates apparently identical by traditional genotyping methods to be distinguished [7], [9]. Fast availability of this precise information on person-to-person transmission to individual healthcare practitioners and institutions is likely to transform the practice of routine infection control [1], [7].

However, potentially undetected mixed infections—infection with two or more unrelated strains of the same species—means that transmission cannot be excluded with complete certainty [10]. This is because if a mixed infection is present in a transmission donor or recipient and only one isolate sampled from each, it is possible the sequenced isolates may differ even though an identical strain is present in both cases. In this scenario, transmission would be incorrectly excluded, exposing a potentially serious weakness of the technique. Even so, sequencing single colonies is common practice in microbiology, and the protocol has underpinned the majority of bacterial WGS studies to date [3]–[8] (but see [11], [12]). Traditional approaches to investigating mixed infection are expensive because they involve separate sub-culture of multiple bacterial colonies, a process in which multiple individual colonies are transferred to a separate culture plate and re-incubated [10]. Because this approach is cost and labour intensive, it is not used in routine clinical laboratories or in large-scale transmission studies.

As WGS in bacteria typically yields generous depth of coverage (measured by the number of reads mapping to any particular site in the sequenced genome [1], [13]), interrogation of these reads offers the prospect of detecting mixed infection by sequencing an aggregate of colonies at the same cost as sequencing an individual colony. In this approach, the short reads produced by next generation sequencers would be mapped to a reference genome using a standard method [13]. The composition of bases mapping to any given nucleotide position can then be analysed to detect evidence of multiple strains. Whereas bacterial genomes should normally be haploid, a pattern of bases that resembles a heterozygous base call in a diploid genome is symptomatic of mixed infection [14]. This idea has been used to detect viral genetic diversity within individual hosts [15], [16]. In viral sequences, the density of single nucleotide polymorphisms (SNPs) may be sufficient to allow common SNPs to be identified between overlapping reads and haplotypes to be reconstructed [15]. Clearly this ability is dependent on the within-host viral diversity, the sequencing technology and depth of sequencing coverage. In contrast, the density of SNPs between sequences in potential mixed bacterial infections is much lower; for example, in the major hospital-associated bacterial pathogen *C. difficile*, there may be 100–10000 SNPs over a total genome of 4.3 million base pairs, which corresponds to just 1 SNP in 400–40000 base pairs [17]. At this density, SNPs are sufficiently sparse that complete haplotype reconstruction is not possible from current short-read sequencing with read lengths of the order of 100 base pairs. Many if not all SNPs are likely to lack adjacent variants closer than the maximum read length, making it impossible to associate these reads with the correct haplotype. The one exception to this is the scenario in which the haplotypes make up markedly different proportions of the sample.

The major healthcare-associated infection, *C. difficile* [18], provides an important example of where undetected mixed bacterial infection may affect estimates of transmission between cases. *C. difficile* causes substantial morbidity and mortality, and is the focus of costly prevention efforts in healthcare systems worldwide [19]. Although it is generally believed that *C. difficile* is predominantly nosocomially acquired [18], a recent study found that <25% of *C. difficile* infections in Oxfordshire, UK, over a 2.5 year period could be linked to a previous case with the same strain type via hospital ward contact [20], suggesting a substantial unsampled reservoir for human infections. However a potential limitation of this study was that only one strain was sequence typed per case and therefore mixed infections could in theory comprise some or much of the unsampled reservoir. *C. difficile* mixed infection rates of ∼7–13% have been consistently described over the last decade [10], [21]–[24], but their significance in transmission has never been investigated. We have therefore developed a method for detecting mixed infection from bacterial WGS data that exploits frequency differences between the dominant and minor strain making up the sample and compares putative base calls to a database of known sequences to assist in determining the haplotypes present. We demonstrate our algorithm performs well in *in silico* and *in vitro* mixed infection experiments and apply it to quantify the extent of transmission arising from mixed *C. difficile* infections in order to determine its relevance to routine hospital outbreak investigations.

## Results

**Figure 1:**
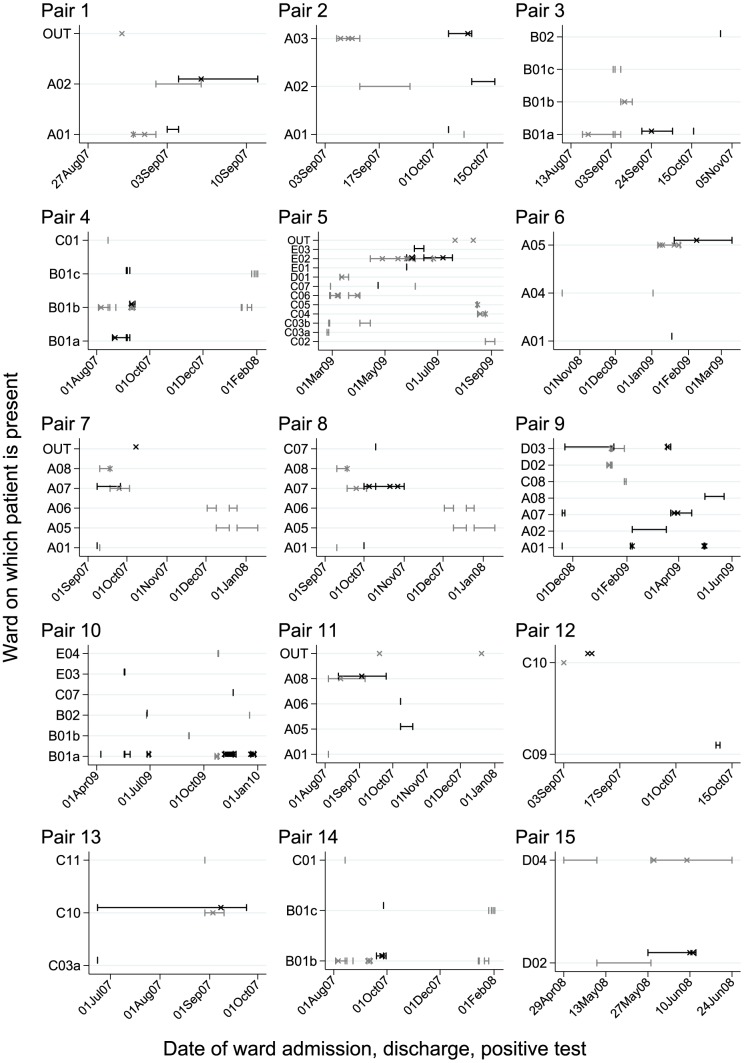
Epidemiological relationships between 15 potential donors and recipients of mixed infection transmission. Potential donors are shown in grey, and potential recipients in black. Time on a hospital ward around the time of diagnosis is shown as a horizontal line bounded by short vertical lines. Each hospital/hospital area is given a distinct letter, each ward a number, and groups of similar wards are given the same number followed by a lower case letter. Positive samples for *C. difficile* are shown as crosses.

Hospital admission and ward movement data on 1276 *C. difficile* infections (CDI) in Oxfordshire, UK (September 2007–March 2010) were analysed in a stochastic compartmental transmission model [25] (see Text S1 for a summary of the model). Low-resolution genetic data was available for each case in the form of multilocus sequence types (MLST) [26], obtained from standard sub-culture of a single bacterial colony [20]. Initially the stochastic transmission model was fitted without incorporating genetic data of any kind (neither MLST nor WGS). From this preliminary analysis, we then cross-referenced to the MLST data in order to identify 15 pairs of cases enriched for likely transmission events associated with mixed infection. Putative transmission between these cases had been inferred by the stochastic transmission model on the basis of close epidemiological linkage, meaning that they either shared space and time on the same hospital ward around the diagnosis of the first case and before the second, or shared the same hospital ward in quick succession such that transmission via highly resistant *C. difficile* spores was possible (Figure 1). All 15 pairs were associated with a high posterior probability of transmission (p>0.45), and had only a single highly likely donor for each recipient. However the single isolate typed from the potential donor and recipient in each pair had different sequence types (STs). As MLST is a low resolution typing method based on sequencing conserved bacterial housekeeping genes, differing STs are likely to be somewhat distinct at the whole genome level, and therefore not compatible with transmission [26]. However, one important and normally overlooked explanation for the ST-mismatch is that the donor or the recipient could have had mixed infection, i.e. that genuine transmission between the two patients was masked by only genotyping one of several strains present in either or both patients. If mixed infection contributes significantly to transmission, it is likely this group of donor-recipient pairs will be enriched for undetected transmissions between multiply infected cases. Therefore these cases form a more sensitive test for mixed infection than random sampling from the total 1276 cases.

Twenty-six faecal samples from 12 putative donors and 15 putative recipients (3 donors had 2 putative recipients, and 1 recipient was also a putative donor) representing all 15 potential mixed-infection transmissions were cultured. DNA was extracted directly from a sweep of multiple colonies taken across each primary culture plate to capture the complete genetic diversity present. Sequencing using the Illumina HiSeq 2000 platform (San Diego, California, USA) generated 100 base-pair reads. Sequence reads were mapped using two aligners, Stampy [27] and Burrows-Wheeler Aligner (BWA) [28] to the *C. difficile* 630 reference genome, CD630 [29].

### Calibration of mixed infection detection

We developed a maximum likelihood based method to detect mixed infection in bacterial WGS data, based on high quality base counts at sites known to vary on the basis of available previously sequenced isolates. The algorithm can be applied to any set of variable sites within a genome. As the stochastic transmission model above had suggested potential mixed ST infections, we initially investigated variable sites within the MLST loci as these are sufficient to demonstrate if mixed ST infection is present. We then investigated sites across the whole genome that are known to vary within an individual ST, allowing us to determine the precise identity of mixed infection strains.

To calibrate the mixed infection estimator all reads from 100 whole genome sequences derived from a single colony, and thus expected not to be mixed, were initially analysed investigating the 150 variable sites within the MLST loci for evidence of mixed ST infection. The ST previously obtained by PCR was recovered using our method on all occasions and accounted for a median of 100% (interquartile range [IQR] 99.9–100%, range 93.5–100%) of the sample based a median (IQR) read depth of 80 (67–93) (Figure S1). The divergence between the dominant and minor haplotypes was estimated at a median 0 SNPs (IQR 0 – 0 SNPs, range 0–15 SNPs). A likelihood ratio statistic was used to compare the maximum likelihood obtained under the mixed infection model, with the likelihood of the data without mixed infection. For each sample in the calibration set we calculated the deviance (−2 times the log likelihood ratio) and used the quantiles of the distribution to set a threshold for calling mixed infection of ≥19.4 in order to achieve a 5% false-positive rate. This empirical approach to choosing the significance threshold avoids making unrealistic assumptions about the statistical distribution of the deviance under the null hypothesis of single infection.

### In silico simulated mixed infection

Simulated mixed infections were generated to test the ability of our method to detect mixed infections, and the constituent strains. Reads obtained from the unmixed samples above were mixed *in silico* to create 10000 simulated mixed ST infections with median (IQR) read depth 78 (67–90) and mixture proportions from 0.5 to 0.95. The known input mixture proportion was estimated with a root mean square error, RMSE, of 0.086 (see Figure S2a for the distribution of estimated mixed proportions across the 10 input proportions). Accurate mixture proportion estimates were obtained even when the simulated sequence divergence was as low as 3 SNPs between dominant and minor sequences. Mixture proportions closer to 1 were associated with a smaller variance and RMSE. The divergence between sequences at the MLST loci was estimated with a RMSE of 0.079, consistently across varying mixture proportions (Figure S2b). Having already set the specificity of the algorithm to 95% with the empirical calibration procedure above, we found that the sensitivity of the algorithm in this dataset for detection of simulated mixed infections was 99.0%.

The two input STs were recovered as the most likely pair on 9704/10000 occasions, 9151 with the correct ordering of the dominant and minor STs (Figure S3). As expected, the minor ST was less likely to be recovered when it made up a smaller proportion of the overall sequence. Recovery of neither input ST was associated with mixture proportions near 0.5 and relatively low divergence between input sequences (median input divergence 0.033 where neither ST recovered versus 0.073 in all other samples, Kruskal–Wallis p<0.001).

### In vitro simulated mixed infection

**Figure 2:**
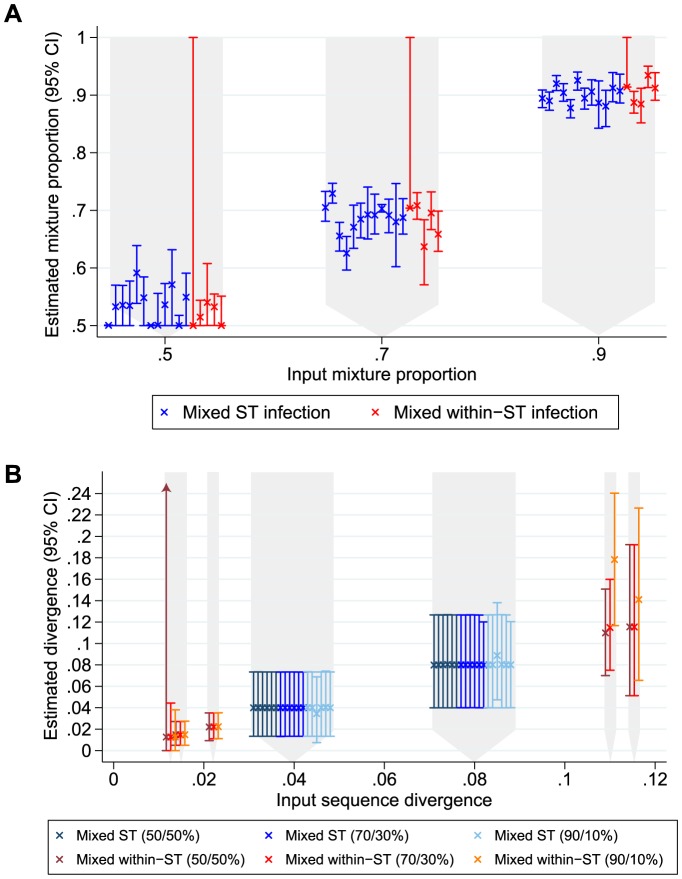
*In vitro* simulated mixed infections. Panel A shows the estimated mixture proportion for 3 input DNA mixture proportions. For ease of visualisation individual data points have different x-axis values, but correspond to the 3 x-axis values as indicated by the grey background. Points obtained from mixes of two differing STs are shown in red, and points from mixing two isolates of the same ST in blue. The large confidence intervals for the leftmost of each red group of samples is a sample with only a single variant site between the two input sequences, which when excluded in bootstrap sampling makes the sample appear unmixed. Panel B shows the estimated divergence between sequences for differing input divergence and mixture proportions. The leftmost group of 3 points represent a sample with a single variant site, which when excluded in bootstrap sampling makes the sample appear unmixed and estimates of d unstable between 0 and 1.

To confirm the performance of the estimator *in vitro*, DNA extracted from single colonies was mixed in known proportions prior to sequencing. Thirty-six mixed ST infections were simulated: DNA from 12 single ST infections was mixed with DNA from 12 different single ST infections, at 3 different mixture proportions – 50/50%, 70/30% and 90/10%. Using our method and whole genome data the input pair of dominant and minor haplotypes was obtained as the most likely on all occasions and the mixture proportion and divergence estimated with RMSEs of 0.032 and 0.002 respectively (Figure 2, supplementary table S1a).

In order to demonstrate our method is also able to detect mixed infections where the two infecting strains are of the same ST, but differ at a whole genome level, we also simulated mixed infections of the same ST. A database of previously sequenced Oxfordshire isolates[30] was used to determine the variable sites within each ST across the rest of the whole genome. These variable sites were analysed for evidence of within-ST mixed infection, using the same algorithm as for mixed ST infection, determining the most likely dominant and minor sequences from the unique whole genome sequences within each ST in the database. Fifteen within-ST mixed infections were generated with DNA from 5 pairs of isolates sharing the same ST (STs 1, 3, 8, 14, 46), but with differing whole genome sequences, at 3 different mixture proportions (50/50%, 70/30% and 90/10%). The correct dominant and minor sequences were obtained on all occasions (Table S1b). Mixture proportions and between sequence divergence were accurately estimated with a single exception where the within sequence divergence was over-estimated in a 90/10% mix (Figure 2). Accurate estimation of mixture proportions was possible even in a mixed infection where the samples differed only by a single site, with estimated mixture proportions of 0.50, 0.70 and 0.92 for input values of 0.50, 0.70 and 0.90. The median (IQR) read depths for these simulations were 82 (72–91).

### Mixed infection transmission samples

Having confirmed the accurate performance of our method, we then applied it to the 15 potential mixed infection transmissions described above where transmission was highly plausible based on hospital contacts but the STs obtained from sequencing single colonies differed (Figure 1).

**Figure 3:**
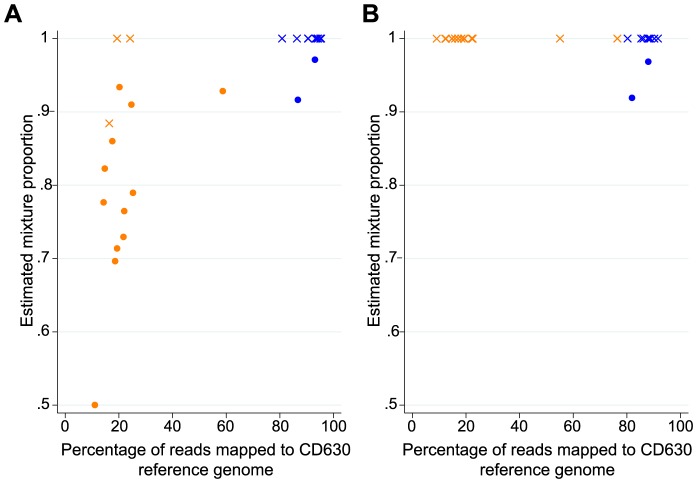
Reads mapped and estimated mixture proportion for possible mixed-ST clinical infections, across two alignment programs. Points in orange show evidence of contamination with other bacteria (i.e. <80% of reads mapped to reference genome), other points are shown in blue. Mixed infections detected using a −2 log likelihood ratio statistic threshold of ≥19.4 (as defined in the calibration samples) are shown as filled circles, other points are shown as crosses. Panel A shows the data obtained from alignments generated using Stampy. Stampy is designed to perform well with relatively high sequence variation relative to the reference, in particular insertions or deletions. In the more contaminated samples we observed markedly divergent reads from other species mapped to the highly conserved MLST loci resulting in falsely identifying mixed infections. Panel B show the data obtained from alignments generated with Burrows Wheeler Aligner. Two mixed infections were detected.

When we aligned sequence data from the 26 samples, the Burrows Wheeler Aligner, BWA [28], outperformed Stampy [27] because a number of samples also included sequence from non-*C. difficile* anaerobic bacteria. DNA for sequencing was obtained from an area of confluent growth on primary culture plates, and despite the use of selective agar and individual colonies resembling *C. difficile*, other similar antibiotic-resistant anaerobic gut bacteria were detected in some samples (by extracting 16S ribosomal RNA genes using BLAST [31] from *de novo* assemblies [32] of the sequences and comparison with the Ribosomal Database Project [33]). Sequence reads from these other species do not map or map poorly to the reference genome, therefore the percentage of reads mapped with Stampy to the CD630 reference ranged from 11.0%–95.4%, with 15/26 samples having <60% of reads mapped. As Stampy is designed to perform well with relatively large sequence variation relative to the reference, in the more contaminated samples markedly divergent reads were mapped to the MLST loci. These reads must have arisen from other species as such divergence would not be expected within the highly conserved housekeeping genes of the MLST loci within *C difficile*. These divergent reads were interpreted by our algorithm as mixed infection, such that a clear relationship was seen between samples estimated to contain mixed infection based on Stampy mapping and those with low percentages of reads mapped to the reference (figure 3a). We therefore remapped all samples with BWA to increase the penalties associated with insertions and deletions relative to the reference such that only reads arising from *C. difficile* would map to the MLST loci. This allowed assessment of the proportion of mixed infection across all 26 samples (figure 3b). Reductions in read depth were modest with BWA compared to Stampy (overall median (IQR) read depth was 21 (15–80) with Stampy across 26 samples, versus 18 (13–75) with BWA).

**Figure 4:**
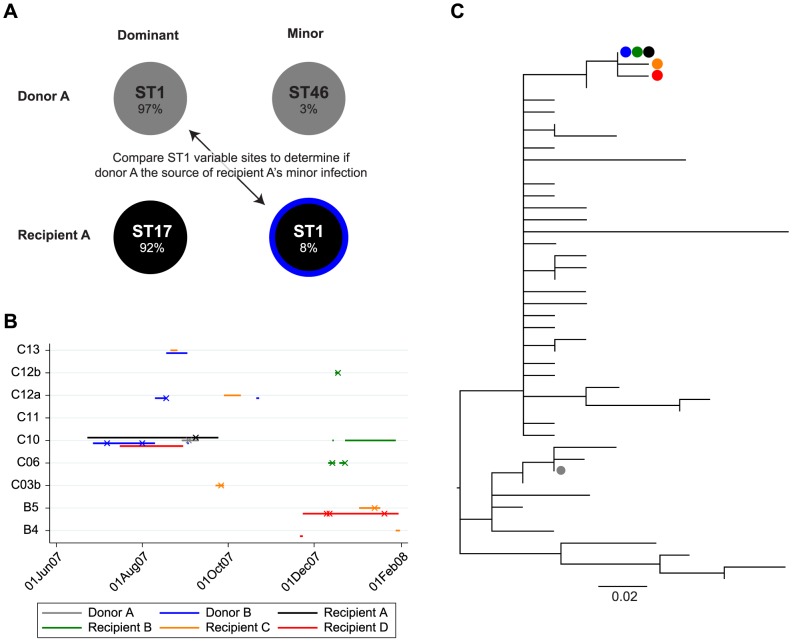
Phylogenetic and epidemiological relationships between cases related to a detected mixed infection. Panel A shows a depiction of the 2 mixed infections identified in donor A and recipient A. A transmission event from donor A to recipient A was predicted by a stochastic transmission model based on ward admission data. However donor A and recipient A had differing multilocus sequence types (STs) on initial testing of a single isolate from each case, suggesting a possible undetected mixed infection. Using the mixed infection estimator a minor ST infection in recipient A was found sharing the same ST, ST1 as donor A. However, applying the estimator to variable sites within ST1, the minor sequence in recipient A was most likely to have arisen from another case, donor B, shown in blue. Panel B shows the epidemiological relationships between donor A, recipient A, donor B and cases sharing similar sequences. Ward stays are shown as horizontal lines and positive tests as crosses. Panel C shows a phylogenetic tree of 45 distinct whole genome sequences from Oxfordshire patients with ST1 *Clostridium difficile* infection. Maximum likelihood tree based on 79 variable sites identified drawn using PhyML [36]. The donor proposed by the transmission model is shown in grey (donor A). The minor sequence in recipient A is shown in black, matching the sequence found in donor B, in blue. Recipient B shared an identical sequence to recipient A. Recipients C and D are two cases phylogenetically descended from the donor B, recipient A, recipient B sequences. Note only donor A and recipient A were analysed for the presence of mixed infection.

Using our method with whole genome data we found 2 of 26 cases (8% of cases, 95% confidence interval, CI, 1–25%) had evidence of mixed ST infection, coincidently in the same transmission model pair, pair 13 (see Figure 1). The estimated dominant ST matched the original ST from MLST PCR in all 26 cases. The putative donor with a mixed ST infection (donor A with a ST1 dominant infection) had a minor ST46 infection that accounted for 3% (95%CI 2–4%) of the sample. However, this did not concord with the dominant ST17 infection found in the putative recipient (recipient A). This recipient in turn had a minor ST recovered, ST1, with sample frequency 8% (95%CI 6–10%), which was compatible with acquisition from donor A. Therefore one of the donor-recipient ST matches predicted by the stochastic transmission model on the basis of shared time and space in the hospital but ruled out by single colony sequencing appeared to be explained by mixed infection (Figure 4).

To scrutinize in more detail whether the WGS data were compatible with transmission of the dominant ST1 infection in donor A to recipient A as a minor infection, we exploited a panel of 45 unique Oxfordshire ST1 genomes to assist in whole genome prediction of the dominant and minor haplotypes in both cases (Figure 4a). Informally, our method compared recipient A's minor ST1 sequence to all 45 ST1 whole genome sequences in our database (which included an ST1 genome sequenced from a single colony from donor A) using a total of 79 ST1-specific SNPs across the whole genome. The most likely recipient A minor sequence (posterior probability = 0.9997) was from another patient (donor B), and differed by 8 SNPs scattered throughout the genome from the sequence found in donor A (Figure 4c). In fact, donor B represents a substantially more plausible donor than donor A identified by the stochastic transmission model on the basis of epidemiological data alone, because the short-term rate of evolution in *C. difficile* has been estimated at ∼1 SNP/genome/year [12], [30]. Donor B was also epidemiologically linked to recipient A, albeit less strongly than donor A. Recipient A was diagnosed on day 77 of a 93-day admission on a surgical ward. Donor B was diagnosed 63 days earlier and spent 34 days after diagnosis on the same ward as the recipient, and was also readmitted for 2 days to the same ward, 6 days before the recipient's diagnosis (Figure 4b). Not only does this reiterate the power of WGS for differentiating potential transmission donors that appear identical on the basis of low-resolution genotyping alone, it also demonstrates that our method is able to extend the approach to mixed infections and identify the source of the minor strain.

We did not find strong evidence for onward transmission from the minor sequence in the mixed infection in recipient A. A single further case (Figure 4b,c, recipient B) with the identical sequence was identified, but the patient had not shared time or space in hospital with donor B or recipient A prior to diagnosis. Given the relatively high prevalence of ST1 and its relatively low diversity even at the whole genome level, indirect transmission via community contact or an undiagnosed third party is the most likely explanation. Additionally, only two descendant sequences (Figure 4, recipient C, recipient D) were identified from the phylogenetic tree of all Oxfordshire ST1s (Figure 4c). Both patients shared time on the same ward with donor B, but not with the mixed infection case (recipient A) after this case's diagnosis. Therefore donor B may have been the source of onward transmissions, but probably not the mixed infection recipient A.

Having found evidence of mixed infections with differing STs, we applied our method to search for previously undetected mixed infections of the same ST in the 24 putative donors and recipients without evidence of mixed-ST infection (Table 1). Five samples contained evidence of mixed infection according to our method. In three cases the divergence estimated between dominant and minor sequences was consistent with levels of within host diversity observed in serially sampled patients where up to 2 SNPs were expected between samples taken on the same day (95% prediction interval) [30]. As such these cases might not have arisen from two transmission events, but from evolution within a host of the same strain. Discounting these 3 cases, we therefore identified 2 mixed infections of the same ST, to add to the 2 mixed infection cases identified with different STs. The two within-ST mixed infection cases had an estimated divergence between the dominant and minor sequences that differed substantially from the best matching sequences in the database, 542 SNPs and 56 SNPs compared to best matches in the database of 1022 SNPs and ≤4 SNPs respectively (Table 1). This suggests the true minor sequence was not present in the database, highlighting our method works best with an established database of local sequences, but is able to identify when novel sequences arise.

**Table 1:** Within ST mixed infection.

Sequence type	Variable sites analysed	Database unique genomes	−2 log likelihood ratio (mixed vs. unmixed infection)	Maximum likelihood estimate mixture proportion, *μ* (95% confidence interval)	Maximum likelihood estimate divergence, *d* in SNPs (95% confidence interval)	Database matched dominant/minor pairs within 95% cumulative probability	SNPs between database-matched dominant and minor pairs
1	78	45	<0.1	1.00 (1.00–1.00)	0.0 (0.0–0.0)		
	79		<0.1	1.00 (1.00–1.00)	0.0 (0.0–0.0)		
	79		<0.1	1.00 (1.00–1.00)	0.0 (0.0–0.0)		
	79		<0.1	1.00 (1.00–1.00)	0.0 (0.0–0.0)		
	79		<0.1	1.00 (1.00–1.00)	0.0 (0.0–0.0)		
**2**	**1613**	**68**	56.8	**0.68 (0.56–1.00)**	**3.1 (0.0–6.4)**	**1**	**6**
	1621		<0.1	1.00 (1.00–1.00)	0.0 (0.0–0.0)		
3	541	38	<0.1	1.00 (1.00–1.00)	0.0 (0.0–0.0)		
	541		<0.1	1.00 (1.00–1.00)	0.0 (0.0–0.0)		
	541		<0.1	1.00 (1.00–1.00)	0.0 (0.0–0.0)		
**5**	**1310**	**41**	568.6	**0.97 (0.96–0.98)**	**541.6 (411.0–763.7)**	**1**	**1022**
**6**	828	65	<0.1	1.00 (1.00–1.00)	0.0 (0.0–0.0)		
	828		<0.1	1.00 (1.00–1.00)	0.0 (0.0–0.0)		
	828		<0.1	1.00 (1.00–1.00)	0.0 (0.0–0.0)		
9	289	19	<0.1	1.00 (1.00–1.00)	0.0 (0.0–0.0)		
10	213	39	15.4	0.95 (0.91–1.00)	8.6 (0.0–34.0)		
16	3012	13	8.4	0.99 (0.97–1.00)	449.4 (32.4–3011.9)		
**17**	**133**	**14**	**133.2**	**0.84 (0.69–1.00)**	**3.8 (0.0–8.5)**	**1**	**41**
	**133**		**75.9**	**0.86 (0.86–1.00)**	**1.2 (0.0–3.8)**	**1**	**69**
18	17	5	<0.1	1.00 (1.00–1.00)	0.0 (0.0–17.0)		
35	586	9	10.8	0.83 (0.83–1.00)	1.0 (0.0–3.0)		
42	163	23	<0.1	1.00 (1.00–1.00)	157.2 (0.0–163.0)		
**44**	**3513**	**55**	**24.4**	**0.98 (0.98–0.99)**	**55.9 (27.0–91.9)**	**13**	**1–4**
63	38	3	<0.1	1.00 (1.00–1.00)	0.0 (0.0–0.0)		

Analysis for mixed infections with two lineages of the same sequence type is shown in 24 donors/recipients without mixed sequence type infection. The number of variable sites within each sequence type contributing to the analysis is shown together with the number of unique sequences of the sequence type present in the database. A likelihood ratio statistic was used to compare the maximum likelihood obtained under the mixed infection model, with the likelihood of the data without mixed infection.

Samples with a −2 log likelihood statistic ≥19.4 were considered mixed (see calibration set results). The probability of each possible dominant/minor sequence pair from the database being the true pair was calculated, and pairs arranged in descending probability. The number of pairs accounting for ≥95% probability is shown in the penultimate column, with the range of SNPs present between the most likely dominant-minor pairs (within 95% cumulative probability) in the last column. This can be compared to the estimated divergence between dominant and minor sequences which is estimated independently and provides a check on whether the pairs identified within the database are consistent with the divergence between the dominant and minor sequences.

## Discussion

We describe a new approach for detecting mixed infection from bacterial whole genome sequence data with low SNP density, utilizing a computationally efficient algorithm that we show performs well in *in silico* and *in vitro* simulations. This offers the prospect of screening for mixed infection in transmission studies and routine outbreak surveillance without labour and cost-intensive individual sub-culture of multiple colony picks and the expense of typing or sequencing these isolates separately. We demonstrate the utility of the approach, which is generalizable to any bacterial pathogen/loci for which a database of known sequences exists, using both WGS and MLST in *C. difficile*.

Our new approach revealed a number of biologically meaningful findings. In our sample of clinical cases significantly enriched for the possibility of mixed ST infection due to the high prior probability of transmission based on epidemiological data but without matching sequence types, we found only 2/26 (8%) cases had evidence of a mixed ST infection. This is consistent with previous estimates for mixed genotype infections of ∼7–13% [10], [21]–[24], although we might have expected to find a higher prevalence had mixed infection genuinely been contributing to transmission. However, these previously undetected mixed infection events could not account for transmission between the 15 putative donor-recipient pairs sequenced. The fact that these pairs were highly selected based on hospital exposure suggests that mixed infection is unlikely to explain a large proportion of the ∼75% CDI cases which cannot be linked to a previous case based on hospital ward exposure [20]. However, by interrogating a larger database of >1200 genomes [30] representing potential donors that were previously sequenced from single colonies, we did find an example of transmission leading to mixed infection. Using the extra resolution afforded by whole genome data we were able to refine our estimate of the likely donor. This revealed a previously undetected transmission event, reflecting additional transmission from the donor, and another transmission event on the ward in question. The significance of the infection for the recipient is unclear; but as ST1 is a virulent strain, it is possible that whilst it only accounted for the minority of the *C. difficile* sequenced it may nevertheless have been the cause of the patient's illness. Therefore, use of our method in outbreak investigation demonstrably offers the ability to detect additional transmission as well as robust determination that true transmission events are not being missed. We were also able to detect mixed infections where both infections shared the same sequence type in 2/24(8%) cases without mixed ST infections and detect likely within host variants in 3 further cases.

In order to capture the full diversity of *C. difficile* present on the primary culture plate a sweep was taken across all the growth. In around half the samples this resulted in contamination of the sequenced reads with other bacterial species, despite morphological appearances consistent with *C. difficile*. This necessitated use of a restrictive mapping algorithm (BWA) favouring mapping reads closely related to the reference. Differences in the proportion of mixed infections estimated by the same algorithm from these two mapping methods highlight the impact of such choices on inferences made from whole genome data. The principle advantage of the primary culture sweep is the ability to capture the full diversity present on the plate, rather than selecting a limited number of colonies for sub-culture. However if contamination with other bacterial species is a concern, one possible refinement still enabling an assessment of mixed infection, without expensive sequencing of multiple single picks, might be to sample multiple individual colonies from the primary culture plate, and sub-culture these together on a single plate prior to sequencing. Paradoxically such approaches may actually increase sensitivity even if only relatively modest numbers of colonies are sampled (e.g. 10–20 colonies). This is because high levels of contamination result in reduced read depths for a given sequencing effort, as evidenced by the lower median depth achieved in samples with <60% of reads mapped, 14, compared to 77 in samples with ≥60% of reads mapped. However, further sub-culture does risk increasing any biases introduced by differential growth of strains on culture media, relative to their original frequency within the host. The sequencing process itself can also potentially introduce read frequency biases, however this did not appear to have a significant impact in the *in vitro* simulations performed.

Although our method assumes mixtures contain only 2 sequences, it still should detect the presence of mixed infection where there is a dominant sequence and several minor sequences. We would expect the estimated minor sequence would be a hybrid of the true minor sequences. This might be apparent where the minor sequence did not match a known sequence, or where >2 nucleotides were found at a single SNP site. A possible hybrid minor sequence could then prompt further more detailed investigation including sub-culture of individual isolates. In the case of *C. difficile*, mixed infection with more than 2 genotypes is reported, but the majority of mixed infections are with 2 genotypes [10], [23]. When comparing bacterial sequences, SNPs are often sparsely distributed throughout the genome, making it likely that a mixture with several minor sequences would still only contain biallelic SNPs. Therefore instead of the two-stage approach of estimating the mixture proportions followed by haplotype matching we demonstrate, any future approach to detect mixtures of more than 2 bacterial sequences would have to jointly estimate mixture proportions and haplotypes. Such an approach would still have to make use of a library of known haplotypes, given the limited numbers of SNPs relative to read lengths. As the current approach also depends on access to a database of known haplotypes this emphasises the benefits of read archives which could enable sequence data generated by different researchers to be incorporated into such a database. Future availability of long read sequencing, including “strand sequencing” with no theoretical read length limit [13], may allow approaches taken in viral sequencing to be applied using SNPs identified at the ends of overlapping sequence fragments to reconstruct haplotypes [15], [16] or may simplify the identification of mixed infection to identifying individual genomes sequenced in a single read. However until then, next-generation whole genome sequencing offers the potential for high-throughput, labour- and cost-effective screening for mixed infection, and such approaches should become the standard when investigating transmission and potential outbreaks.

## Methods

### Ethics statement

This study was approved by the Berkshire Research Ethics Committee (10/H0505/83) and the National Information Governance Board (8-05(e)/2010) without requiring individual patient consent.

### Sample preparation and sequencing

Selective culture for *C. difficile* was undertaken following an alcohol-shock on modified Brazier's cycloserine-cefoxitin-egg yolk agar. Plates were incubated anaerobically at 37°C for up to 7 days following the method of Griffiths *et al* [26]. DNA was extracted directly from a sweep taken across each primary culture plate to capture the complete genetic diversity present: a 5 µl loop was passed through an area of confluent growth, and the loopful of growth then suspended in saline prior to DNA extraction with a commercial kit (QuickGene, Fujifilm, Tokyo, Japan). All growth was morphologically consistent with *C. difficile*, exhibited a characteristic odour and fluoresced under ultraviolet light. Extracted DNA underwent whole genome sequencing using the Illumina HiSeq 2000 platform (San Diego, California, USA) generating 100 base-pair reads. Sequence reads were mapped using two aligners, Stampy [27] (with an expected substitution rate of 0.01) and Burrows-Wheeler Aligner (BWA, with default settings) [28] to the *C. difficile* 630 reference genome (Genbank:AM180355), CD630 [29]. High quality base counts were extracted from mapped data for variable sites using SAMtools [34], retaining bases with a base quality score ≥30 and a mapping quality score ≥30. As the initial algorithm was designed to detect mixed ST infection, the variable sites analysed were first restricted to the 150 single nucleotide variants (SNPs) within the 7 MLST loci based on all published alleles [35]. The variable sites studied were subsequently extended to make full use of the whole genome data, see results above. To allow extraction of 16S ribosomal RNA genes, reads were also assembled *de novo* using Velvet with the Velvet Optimiser [31].

### Estimation of mixture proportion and haplotype divergence

Each sample was assumed to be a mixture of 2 haplotypes, resulting in one dominant and one minor haplotype, with the proportion of the total sequence present made up by the dominant haplotype denoted *μ*. For each sample analysed we let,

N = total number of variable sites considered

*n_j_* = total number of reads at a variable site *j* = 1…N

*b_ij_* = an observed nucleotide from a single read *i* = 1…*n_j_* mapped to variable site *j*, from the set {A, C, G, T}

*B_j_* = a vector of the *n_j_* nucleotides from the reads mapped to site *j*, 
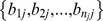


*ε* = Pr(sequencing error in a base call). Assumed constant across all bases calls, having filtered our data to exclude low quality bases and reads

*μ* = proportion of the sample from the dominant haplotype (0.5≤*μ≤1)*

*a_1j_* = nucleotide in the dominant haplotype at site *j*

*a_2j_* = nucleotide in the minor haplotype at site *j*

*A_j_* = the combination of nucleotides in the dominant and minor haplotypes respectively, *a_1j_* and *a_2j_*, one of the set of all 16 possible pairs of nucleotides, *A*:
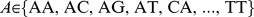
(1)We expressed the probability of observing a particular nucleotide in a given read mapped to site *j* in terms of the underlying dominant and minor haplotypes, the mixture proportion and error probability. We assume the probability of a sequence error, *ε*, is constant across all variable sites, and if an error occurs it is equally likely to result in any of the three alternative nucleotides (i.e. if the true nucleotide is A, then a read containing C, G, or T is equally likely):
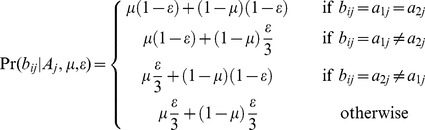
(2)As *ε* is treated as a known constant, the probability of observing the *n_j_* nucleotides mapped to site *j*, for a given value of *A_j_* is:
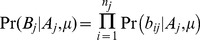
(3)Where *A_j_* is unknown, summing over all possible values of *A_j_* gives:
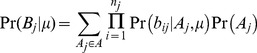
(4)To define Pr(*A_j_*) for each possible value of *A_j_* we let *d* be the proportion of all variable sites included in the analysis that are divergent between the dominant and minor haplotypes. At sites divergent between the haplotypes 12 possible pairs of nucleotides could be present, and at non-divergent sites 4 pairs of nucleotides are possible, such that:
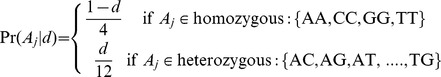
(5)Combining (4) and (5), we then expressed the probability of observing the *n_j_* nucleotides mapped to site *j*, for a given mixture proportion and divergence between the dominant and minor haplotypes:
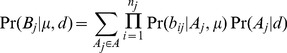
(6)The values of *μ* and *d* were then jointly estimated from their likelihood:
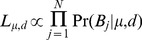
(7)A major question is how to determine whether the data are consistent with a mixed infection. A simple but arbitrary approach would be fix thresholds of *μ* and *d*, (e.g. 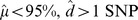). However, we adopted an alternative approach: comparing the maximum likelihood obtained under the mixed infection model with the likelihood of the data without mixed infection, i.e. *μ* = 1, *d* = 0. Comparing the log-likelihood ratio statistic to a chi-squared distribution is problematic, as the null hypothesis is on the edge of the parameter space. Therefore we used a calibration set of 100 samples known not to contain mixed infection to determine a deviance (−2 log likelihood ratio) threshold for confirming mixed infection. The value of the threshold was chosen to achieve a 5% false positive rate. This empirical approach by using a calibration set of actual sequences also has the advantage that it accounts for low-level sample contamination that may occur during sequencing. This would not be easily accounted for in another alternative for determining mixed infection, simulating under the null hypothesis in a bootstrapping approach.

Confidence intervals for *μ* and *d* were generated by non-parametric bootstrap sampling. The variable sites were sampled with replacement 1000 times keeping each *B_j_* constant.

### Estimation of haplotypes

A database of known sequences was used to estimate the dominant and minor haplotypes present, using the estimate of *μ* obtained above. From (6), for each potential haplotype pair the value of *A_j_* and *d* are known therefore the probability of observing all the nucleotides mapped to site *j* is:
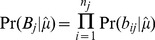
(8)Therefore the likelihood of a given pair of dominant and minor haplotypes given the sequence data is:
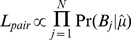
(9)Finally, the posterior probability of each pair of haplotypes was obtained using the product of the prevalence of the dominant and minor haplotypes as their prior probability and the likelihood of each pair above. For MLST analyses the prevalence of each ST in Oxfordshire from September 2007 to March 2010 was used [20]. STs not found during the study but present in pubMLST [35] were assumed to be as prevalent as the least common ST. For whole genome analyses the prevalence of each unique sequence during the study was used. When using whole genome data to assess whether the dominant and minor pair selected were consistent with the data the estimated divergence and 95% confidence intervals were compared with the actual divergence between the dominant and minor sequences. Where the actual pairwise divergence fell within the estimated 95% confidence interval the pair was considered a good match. However when the divergence fell outside of the confidence interval this was interpreted as evidence that the true dominant or minor sequence was not present in the database.

For all analyses the value of *ε* was set to the sum of the base and error mapping rates, assuming each had a PHRED score of 30 (the thresholds used for determining high quality bases to retain in the analysis), i.e. *ε* = 2×10^−3^. This represents the upper bound on the value of *ε* after filtering. However results were similar with lower values of *ε* tested up to *ε* = 2×10^−4^.

To generate *in silico* simulated mixed infections two samples known not to be mixed themselves were mixed in varying proportions. Firstly, read depths were normalised across the two samples by multiplying base counts in the sample with lower coverage to match the coverage in the other sample. Nucleotides were then sampled from reads mapped to each variable site (with replacement). The input sequence each read was sampled from was determined using a binomial distribution with parameters of normalised read depth, and input mixture proportion.

All analyses were conducted using R (http://www.r-project.org). The code used can be found in Text S2. Text S3 contains a short Python (http://www.python.org) script that can be used to obtain high quality base counts from mapped BAM files. Text S4 contains an explanation of the required input files for Text S2 and the output generated. Dataset S1 contains an example dataset of the 26 patient samples analysed for the presence of mixed ST infection, and provides an example of the formatting on the input and output files.

### Data sharing

The sequences reported in this paper have been deposited in the European Nucleotide Archive Sequence Read Archive under study accession number ERP002428 and are available at http://www.ebi.ac.uk/ena/data/view/ERP002428.

## Supporting Information

Dataset S1 **Sample dataset, 26 patient samples analysed for mixed ST infection.** Example input and output files for Text S2.
(ZIP)

Figure S1 **Estimated mixture proportion and sequence divergence for 100 sequences derived from a single colony.** Markers are weighted by frequency. A likelihood ratio test was used to compare the maximum likelihood obtained under the mixed infection model, with the likelihood of the data without mixed infection. For each sample in the calibration set we calculated −2 times the log likelihood ratio and used the quantiles of the distribution to set a threshold for calling mixed infection of ≥19.4 in order to approximate a 5% false-positive rate. Under the model the mixture proportion, between sequence divergence and the read depth contribute to the likelihood of a mixed infection, hence the pattern seen in the red, false-positive, dots where lower mixture proportions, increased sequence divergence, and increased read depth (not shown) contribute to a significant result.
(EPS)

Figure S2 **Estimation of the mixture proportion and sequence divergence across simulated mixtures.** Panel A shows distribution of estimated mixture proportions over 1000 different pairs of input sequences, separately for 10 different input mixture proportions. The estimated mixture proportion shown is for the input dominant sequence. Panel B shows the relationship between the true proportion of sites divergent and the estimated proportion for the same simulations. RMSE, root mean square error.
(EPS)

Figure S3 **Estimation of dominant and minor ST across simulated mixtures.** Estimation based on analysis of 150 variable sites within the MLST loci. At input mixture proportions of 0.5 either order of dominant and minor ST was considered correct.
(EPS)

Table S1 **Estimated mixture proportion and sequence divergence for 51** ***in vitro*** **mixed infections.** DNA from two previously sequenced isolates of differing sequence types (ST) was mixed in 3 proportions 50/50%, 70/30%, 90/10% for 12 pairs of isolates to created 36 mixed ST infections (panel A). DNA from two previously sequenced isolates of the same sequence type was mixed in the same proportions for 5 pairs of isolates to create 15 within-ST mixed infections (panel B). At input mixture proportions of 0.5 either order of dominant and minor ST was considered correct. A likelihood ratio statistic was used to compare the maximum likelihood obtained under the mixed infection model, with the likelihood of the data without mixed infection. Samples with a −2 log likelihood ratio ≥19.4 were considered mixed (see calibration set results).
(DOC)

Text S1 **Stochastic transmission model description.** A description of the stochastic transmission model used to identify potential transmission events arising from mixed infection.
(DOC)

Text S2 **Mixed infection estimator code.** R code implementing the mixed infection estimator algorithm.
(TXT)

Text S3 **High quality base count extractor.** Python code to extract high quality base counts from mapped BAM files is provided. Generates output in the required format for Text S2.
(TXT)

Text S4 **Mixed infection estimator readme file.** A description of the required input files for Text S2. Details of an example dataset, Dataset S1, are provided.
(DOC)
