# Supplementary material for: Detection of Mixed Infection from Bacterial Whole Genome Sequence Data Allows Assessment of Its Role in Clostridium difficile Transmission
Source: PLoS Comput Biol. 2013 May 2;9(5):e1003059. doi: 10.1371/journal.pcbi.1003059 (PMC3642043; doi:10.1371/journal.pcbi.1003059)
Supplement: Table S1 — Estimated mixture proportion and sequence divergence for 51 in vitro mixed infections. DNA from two previously sequenced isolates of differing sequence types (ST) was mixed in 3 proportions 50/50%, 70/30%, 90/10% for 12 pairs of isolates to created 36 mixed ST infections (panel A). DNA from two previously sequenced isolates of the same sequence type was mixed in the same proportions for 5 pairs of isolates to create 15 within-ST mixed infections (panel B). At input mixture proportions of 0.5 either order of dominant and minor ST was considered correct. A likelihood ratio statistic was used to compare the maximum likelihood obtained under the mixed infection model, with the likelihood of the data without mixed infection. Samples with a −2 log likelihood ratio ≥19.4 were considered mixed (see calibration set results). (DOC) [file pcbi.1003059.s005.doc]

**Supplementary table**

**A**

| **Type** | **ST sample 1** | **ST sample 2** | **Input *d*, SNPs** | **Input *µ*** | **Maximum likelihood estimate *µ* (95% confidence interval)** | **Maximum likelihood estimate *d* in SNPs (95% confidence interval)** | **-2 log likelihood ratio (mixed vs. unmixed infection)** | **Estimated probability of input pair** |
| --- | --- | --- | --- | --- | --- | --- | --- | --- |
| Mixed-ST infection | 1 | 2 | 12 | 0.5 | 0.50 (0.50 - 0.50) | 12.0 (6.0 - 19.0) | 6239.8 | 1.000 |
| Mixed-ST infection | 14 | 67 | 12 | 0.5 | 0.53 (0.50 - 0.57) | 12.0 (6.0 - 19.0) | 4515.1 | 1.000 |
| Mixed-ST infection | 1 | 44 | 12 | 0.5 | 0.54 (0.50 - 0.57) | 12.0 (6.0 - 19.0) | 3275.3 | 0.997 |
| Mixed-ST infection | 1 | 16 | 12 | 0.5 | 0.53 (0.50 - 0.58) | 12.1 (6.0 - 19.0) | 4537.8 | 1.000 |
| Mixed-ST infection | 1 | 44 | 12 | 0.5 | 0.59 (0.54 - 0.64) | 12.0 (6.0 - 19.0) | 3209.6 | 1.000 |
| Mixed-ST infection | 1 | 2 | 12 | 0.5 | 0.55 (0.50 - 0.58) | 12.0 (6.0 - 19.0) | 3392.6 | 1.000 |
| Mixed-ST infection | 34 | 33 | 6 | 0.5 | 0.50 (0.50 - 0.50) | 6.0 (2.0 - 11.0) | 2136.4 | 1.000 |
| Mixed-ST infection | 14 | 44 | 6 | 0.5 | 0.50 (0.50 - 0.56) | 6.0 (2.0 - 11.0) | 2487.8 | 1.000 |
| Mixed-ST infection | 8 | 13 | 6 | 0.5 | 0.54 (0.50 - 0.57) | 6.0 (2.0 - 11.0) | 2476.4 | 1.000 |
| Mixed-ST infection | 14 | 44 | 6 | 0.5 | 0.57 (0.50 - 0.63) | 6.0 (2.0 - 11.0) | 2828.1 | 1.000 |
| Mixed-ST infection | 8 | 49 | 6 | 0.5 | 0.50 (0.50 - 0.52) | 6.0 (2.0 - 11.0) | 3639.6 | 0.333 |
| Mixed-ST infection | 2 | 54 | 6 | 0.5 | 0.55 (0.50 - 0.59) | 6.0 (2.0 - 11.0) | 3423.9 | 0.958 |
| Mixed-ST infection | 2 | 1 | 12 | 0.7 | 0.71 (0.68 - 0.73) | 12.0 (6.0 - 19.0) | 3444.5 | 1.000 |
| Mixed-ST infection | 67 | 14 | 12 | 0.7 | 0.73 (0.71 - 0.75) | 12.0 (6.0 - 19.0) | 3417.9 | 1.000 |
| Mixed-ST infection | 44 | 1 | 12 | 0.7 | 0.66 (0.63 - 0.68) | 12.0 (6.0 - 19.0) | 3668.2 | 1.000 |
| Mixed-ST infection | 16 | 1 | 12 | 0.7 | 0.63 (0.60 - 0.65) | 12.0 (6.0 - 19.0) | 3483.5 | 1.000 |
| Mixed-ST infection | 44 | 1 | 12 | 0.7 | 0.67 (0.63 - 0.71) | 12.0 (6.0 - 19.0) | 2918.8 | 1.000 |
| Mixed-ST infection | 2 | 1 | 12 | 0.7 | 0.68 (0.65 - 0.71) | 12.0 (6.0 - 18.0) | 3401.8 | 1.000 |
| Mixed-ST infection | 33 | 34 | 6 | 0.7 | 0.69 (0.65 - 0.74) | 6.0 (2.0 - 11.0) | 1847.1 | 1.000 |
| Mixed-ST infection | 44 | 14 | 6 | 0.7 | 0.69 (0.66 - 0.73) | 6.0 (2.0 - 11.0) | 1784.5 | 1.000 |
| Mixed-ST infection | 13 | 8 | 6 | 0.7 | 0.70 (0.70 - 0.71) | 6.0 (2.0 - 11.0) | 1305.9 | 1.000 |
| Mixed-ST infection | 44 | 14 | 6 | 0.7 | 0.69 (0.66 - 0.72) | 6.0 (2.0 - 11.0) | 2326.4 | 1.000 |
| Mixed-ST infection | 49 | 8 | 6 | 0.7 | 0.68 (0.60 - 0.75) | 6.0 (2.0 - 11.0) | 1553.4 | 1.000 |
| Mixed-ST infection | 54 | 2 | 6 | 0.7 | 0.69 (0.66 - 0.72) | 6.0 (2.0 - 11.0) | 2120.8 | 1.000 |
| Mixed-ST infection | 1 | 2 | 12 | 0.9 | 0.89 (0.88 - 0.91) | 12.0 (6.0 - 19.0) | 1112.4 | 1.000 |
| Mixed-ST infection | 14 | 67 | 12 | 0.9 | 0.89 (0.87 - 0.91) | 12.0 (6.0 - 19.0) | 866.4 | 1.000 |
| Mixed-ST infection | 1 | 44 | 12 | 0.9 | 0.92 (0.91 - 0.93) | 13.3 (7.1 - 20.7) | 526.0 | 1.000 |
| Mixed-ST infection | 1 | 16 | 12 | 0.9 | 0.90 (0.89 - 0.92) | 12.1 (6.0 - 19.0) | 785.2 | 1.000 |
| Mixed-ST infection | 1 | 44 | 12 | 0.9 | 0.88 (0.86 - 0.89) | 12.0 (6.0 - 19.0) | 1072.7 | 1.000 |
| Mixed-ST infection | 1 | 2 | 12 | 0.9 | 0.93 (0.91 - 0.94) | 12.0 (6.0 - 18.1) | 576.1 | 0.995 |
| Mixed-ST infection | 34 | 33 | 6 | 0.9 | 0.89 (0.88 - 0.91) | 6.0 (2.0 - 11.0) | 382.4 | 1.000 |
| Mixed-ST infection | 14 | 44 | 6 | 0.9 | 0.91 (0.88 - 0.93) | 6.0 (2.0 - 11.0) | 401.5 | 1.000 |
| Mixed-ST infection | 8 | 13 | 6 | 0.9 | 0.89 (0.84 - 0.92) | 5.2 (1.1 - 10.3) | 317.5 | 1.000 |
| Mixed-ST infection | 14 | 44 | 6 | 0.9 | 0.88 (0.85 - 0.91) | 6.0 (2.0 - 11.0) | 540.9 | 1.000 |
| Mixed-ST infection | 8 | 49 | 6 | 0.9 | 0.91 (0.89 - 0.94) | 6.1 (2.0 - 11.1) | 228.5 | 1.000 |
| Mixed-ST infection | 2 | 54 | 6 | 0.9 | 0.91 (0.89 - 0.94) | 6.0 (2.0 - 11.0) | 360.1 | 1.000 |

**B**

| **Type** | **ST sample 1** | **ST sample 2** | **Input *d*, SNPs** | **Input *µ*** | **Maximum likelihood estimate *µ* (95% confidence interval)** | **Maximum likelihood estimate *d* in SNPs (95% confidence interval)** | **-2 log likelihood ratio**  **(mixed vs. unmixed infection)** | **Estimated probability of input pair** |
| --- | --- | --- | --- | --- | --- | --- | --- | --- |
| Within-ST mixed infection | 1 | 1 | 1 | 0.5 | 0.50 (0.50 - 1.00) | 1.0 (0.0 - 78.7) | 510.7 | 1.000 |
| Within-ST mixed infection | 3 | 3 | 12 | 0.5 | 0.51 (0.50 - 0.54) | 11.9 (5.0 - 19.0) | 4874.9 | 1.000 |
| Within-ST mixed infection | 8 | 8 | 6 | 0.5 | 0.54 (0.50 - 0.61) | 6.0 (2.0 - 11.0) | 1695.9 | 0.995 |
| Within-ST mixed infection | 14 | 14 | 22 | 0.5 | 0.53 (0.50 - 0.55) | 22.0 (14.0 - 30.2) | 11975.4 | 1.000 |
| Within-ST mixed infection | 46 | 46 | 9 | 0.5 | 0.50 (0.50 - 0.55) | 9.0 (4.0 - 15.0) | 3374.6 | 1.000 |
| Within-ST mixed infection | 1 | 1 | 1 | 0.7 | 0.70 (0.70 - 1.00) | 1.0 (0.0 - 3.5) | 323.9 | 1.000 |
| Within-ST mixed infection | 3 | 3 | 12 | 0.7 | 0.71 (0.68 - 0.73) | 12.0 (6.0 - 19.0) | 3298.9 | 1.000 |
| Within-ST mixed infection | 8 | 8 | 6 | 0.7 | 0.64 (0.57 - 0.68) | 6.0 (2.0 - 11.0) | 1631.9 | 1.000 |
| Within-ST mixed infection | 14 | 14 | 22 | 0.7 | 0.70 (0.67 - 0.73) | 23.0 (15.0 - 32.0) | 6839.6 | 1.000 |
| Within-ST mixed infection | 46 | 46 | 9 | 0.7 | 0.66 (0.63 - 0.70) | 9.0 (4.0 - 15.0) | 2760.8 | 1.000 |
| Within-ST mixed infection | 1 | 1 | 1 | 0.9 | 0.91 (0.91 - 1.00) | 1.0 (0.0 - 3.0) | 57.6 | 1.000 |
| Within-ST mixed infection | 3 | 3 | 12 | 0.9 | 0.89 (0.87 - 0.91) | 12.1 (6.0 - 19.1) | 841.6 | 1.000 |
| Within-ST mixed infection | 8 | 8 | 6 | 0.9 | 0.88 (0.85 - 0.91) | 6.0 (2.0 - 11.1) | 279.4 | 1.000 |
| Within-ST mixed infection | 14 | 14 | 22 | 0.9 | 0.93 (0.91 - 0.95) | 35.7 (23.3 - 48.1) | 1136.4 | 1.000 |
| Within-ST mixed infection | 46 | 46 | 9 | 0.9 | 0.91 (0.89 - 0.94) | 11.0 (5.1 - 17.7) | 488.9 | 1.000 |

**Table S1. Estimated mixture proportion and sequence divergence for 51 *in vitro* mixed infections.** DNA from two previously sequenced isolates of differing sequence types (ST) was mixed in 3 proportions 50/50%, 70/30%, 90/10% for 12 pairs of isolates to created 36 mixed ST infections (panel A). DNA from two previously sequenced isolates of the same sequence type was mixed in the same proportions for 5 pairs of isolates to create 15 within-ST mixed infections (panel B). At input mixture proportions of 0.5 either order of dominant and minor ST was considered correct. A likelihood ratio statistic was used to compare the maximum likelihood obtained under the mixed infection model, with the likelihood of the data without mixed infection. Samples with a -2 log likelihood ratio ≥19.4 were considered mixed (see calibration set results).
