# Supplementary material for: Detection of Mixed Infection from Bacterial Whole Genome Sequence Data Allows Assessment of Its Role in Clostridium difficile Transmission
Source: PLoS Comput Biol. 2013 May 2;9(5):e1003059. doi: 10.1371/journal.pcbi.1003059 (PMC3642043; doi:10.1371/journal.pcbi.1003059)
Supplement: Text S1 — Stochastic transmission model description. A description of the stochastic transmission model used to identify potential transmission events arising from mixed infection. (DOC) [file pcbi.1003059.s006.doc]

**Stochastic transmission model**

The dataset analysed in the stochastic transmission model is described in more detail here [1]. For each patient diagnosed with *C. difficile* infection during the study, data were available on hospital admissions and ward movements, date of diagnosis and the multi-locus sequence type of the infecting isolate. Data were also available on hospital admissions and ward movements for all hospital inpatients not diagnosed with *C. difficile* allowing the number of susceptible patients on each ward to be accounted for in the model.

Briefly, a stochastic compartmental model was analysed in a Bayesian framework using Markov Chain Monte Carlo [2]. Patients were modelled as susceptible, colonised, infectious, or recovered. At the start of the study all individuals were assumed to be susceptible. Within the model colonised patients carry *C. difficile*, but are not infectious. The use of a colonised state allows the model to account for the period between acquiring *C. difficile* and the start of symptomatic infection, known to be almost always triggered by antibiotic exposure [3]. Although information on antibiotic exposure was not available in the dataset analysed, differing rates of transition from colonised to infectious were included in the model depending on whether patients were currently admitted to hospital or in the community. This allows the increased antibiotic exposure patients are likely to have received in hospital to be accounted for. The date of diagnosis provides information about the likely onset of symptoms and therefore infectiousness, however the model allows for a period of pre-test infectiousness to account for the lag between the onset of symptoms and each cases’ diagnosis.

Transition from susceptible to colonised was determined by differential infection pressure from patients sharing the same ward, sharing the same hospital, and also from recently discharged patients who may have left behind residual ward contamination. Patients were assumed to mix homogenously within a ward, such that an infectious patient is equally likely to infect any susceptible patient on a ward. In addition a background infectious pressure was included to account for transmissions not arising from a known symptomatic case. As mentioned, transition from colonised to infectious occurred at different rates in the community and in hospital to reflect differing antibiotic pressures in each location. Two different recovery rates were included to allow for the minority of patients who develop on-going or recurrent disease.

Model outputs include posterior distributions on model parameters such as transition rates between states, but importantly for the analysis presented in this manuscript, also include estimates of the likely sources of each infection. From the model fifteen pairs of cases were identified with a high posterior probability of transmission between them, but discordant multi-locus sequence types. One possible explanation is that the transmission donor or the recipient could have had mixed infection, i.e. that genuine transmission between the two patients was masked by only having genotyped one of several strains present in either or both patients. If mixed infection contributes significantly to transmission, it is likely this group of donor-recipient pairs will be enriched for undetected transmissions between multiply infected cases. These cases therefore form the basis for this study.

**References**

1. Walker AS, Eyre DW, Wyllie DH, Dingle KE, Harding RM, et al. (2012) Characterisation of Clostridium difficile hospital ward-based transmission using extensive epidemiological data and molecular typing. Plos Medicine 9: e1001172:1–e1001172:12. doi:10.1371/journal.pmed.1001172.

2. Cule ML, Bowden R, Eyre DW, Walker AS, Griffiths D, et al. (2012) The biology and epidemiology of *Clostridium difficile* in Oxfordshire hospitals 2007-2010. In the program and abstracts of ID Week, San Diego, 2012.

3. Cohen SH, Gerding DN, Johnson S, Kelly CP, Loo VG, et al. (2010) Clinical practice guidelines for Clostridium difficile infection in adults: 2010 update by the society for healthcare epidemiology of America (SHEA) and the infectious diseases society of America (IDSA). Infect Control Hosp Epidemiol 31: 431–455. doi:10.1086/651706.
