# Supplementary material for: Detection of Mixed Infection from Bacterial Whole Genome Sequence Data Allows Assessment of Its Role in Clostridium difficile Transmission
Source: PLoS Comput Biol. 2013 May 2;9(5):e1003059. doi: 10.1371/journal.pcbi.1003059 (PMC3642043; doi:10.1371/journal.pcbi.1003059)
Supplement: Text S4 — Mixed infection estimator readme file. A description of the required input files for Text S2. Details of an example dataset, Dataset S1, are provided. (DOC) [file pcbi.1003059.s009.doc]

**Mixed infection estimator**

***Summary***

An algorithm for detection of mixed infections in bacterial whole genome sequence data. The algorithm analyses a set of defined variable sites for evidence of mixed infection with up to 2 strains, if a mixed infection is present, the relative proportions of the dominant and minor haplotypes are estimated. A database of known haplotypes is used to identify the most likely dominant and minor haplotype.

***Dependencies***

Text_S2.R has been tested on R 2.15 on Mac, Windows and Ubuntu. It depends on the foreach library. Support for running the bootstrap part of the algorithm on multiple cores is available on Mac and Linux platforms via the doMC library. On Windows the operating system is detected and a single core used. It is suggested that output buffering is disabled on Windows (via the Misc menu in RGui) to allow monitoring of the script’s progress.

***Required input files***

A copy of the code implementing our algorithm is supplied as Text_S2.R. Several input files are required and are detailed in the code’s annotation. The files are listed below:

1. A database of haplotypes to match to, supplied as a list of haplotypes, as a tab delimited file with 2 columns and no header. Each row should contain the haplotype identifier and secondly the haplotype sequence as a continuous string
2. A list of the relative population frequencies of each haplotype, supplied as a tab delimited file with 2 columns and no header, firstly the identifier, then the frequency. Where haplotype frequencies are unknown the algorithm can still be applied if the frequency of each haplotype is set to 1/N_h_, where N_h_ is the number of haplotypes
3. A file containing a list of the file names for the files containing the base count data, with no header, and a new line for each file name
4. The base counts for each sample are supplied as a separate tab delimited file with the following 5 columns: site, A, C, G, T. Column headings should be included. A separate line is included for each site, and then an integer value for the number of each nucleotides, e.g. for site 113424:

113424 0 0 98 2

To assist generation of base count files from mapped BAM files an annotated python script using pySAM to access SAMTools is provided, Text_S3.py.

***Example dataset***

To provide an example of the required input files Dataset_S1.zip is provided. This archive contains the following files which correspond to the numbered items in the required input files section:

1. haplotype_sequence.txt – a list of haplotypes, containing identifiers and sequence; in this case the concatenated *C. difficile* multilocus sequence typing variable sites
2. haplotype_frequency.txt – a list of the relative population frequency of the known haplotypes; in this case the Oxfordshire 2007-2010 population frequencies of the multilocus sequence types obtained from *C. difficile* infections
3. filenames_list.txt – a list of filenames for the files containing the base count data, see 4)
4. 26 files with filenames in the format C0000XXXX_mlst_bc.txt – files containing the base count data for the 26 samples analysed in the paper for evidence of mixed infection
5. sample_output.txt – this file contains the output from running Text_S2.R on the example dataset

***Outputs***

The output file has the following headings and contents:

- id, the file name containing the base count data analysed, each row of the output file, corresponds to one input base counts file
- ML_m, the maximum likelihood estimate of the mixture proportion, *µ*
- ML_d, the maximum likelihood estimate of the sequence divergence between the dominant and minor haplotypes, *d*
- deviance, the deviance statistic calculated to determine if mixed infection is present, values above the deviance threshold indicate mixed infection is present
- m0.025, the lower bound of the 95% confidence interval for the value of *µ*, calculated by non-parametric bootstrap
- m0.975, the upper bound of the 95% confidence interval for the value of *µ*, calculated by non-parametric bootstrap
- d0.025, the lower bound of the 95% confidence interval for the value of *d*, calculated by non-parametric bootstrap
- d0.975, the upper bound of the 95% confidence interval for the value of *d*, calculated by non-parametric bootstrap
- p_haplotype_pair, for samples where mixed infection is detected, the most likely haplotype pairs are reported in the format pair_name_1: posterior_probability_1, pair_name2: posterior_probability_2, etc

david.eyre@ndm.ox.ac.uk

04 March 2013
